# Association between serum cystatin C and early impairment of cardiac function and structure in type 2 diabetes patients with normal renal function

**DOI:** 10.1002/clc.23920

**Published:** 2022-09-14

**Authors:** Zhuoshan Huang, Junlin Zhong, Shaozhao Zhang, Zhenyu Xiong, Yiquan Huang, Menghui Liu, Yifen Lin, Xiangbin Zhong, Xiaomin Ye, Xiaodong Zhuang, Xinxue Liao

**Affiliations:** ^1^ Department of Cardiovascular Medicine, The Third Affiliated Hospital Sun Yat‐sen University Guangzhou China; ^2^ Cardiology Department, First Affiliated Hospital Sun Yat‐Sen University Guangzhou China; ^3^ NHC Key Laboratory of Assisted Circulation Sun Yat‐Sen University Guangzhou China; ^4^ Department of Ultrasonography, The Third Affiliated Hospital Sun Yat‐sen University Guangzhou China

**Keywords:** cardiac structure, cystatin C, impaired left ventricular diastolic function, left ventricular hypertrophy, type 2 diabetes

## Abstract

**Background:**

Type 2 diabetes mellitus (T2DM) patients may have cardiac remodeling and dysfunction from the early stage of disease. This study aimed to determine the association between cystatin C (CysC) and early cardiac functional or structural impairment in T2DM patients without renal dysfunction.

**Methods:**

A total of 1135 T2DM patients without renal dysfunction and known heart diseases were included in our study. Cardiac function and structure were evaluated by echocardiography. Patients were diagnosed as left ventricular hypertrophy (LVH), impaired left ventricular (LV) diastolic function, and categorized into four different LV geometry patterns including normal, concentric remodeling, concentric hypertrophy, and eccentric hypertrophy.

**Results:**

In multivariate linear regression analyses, CysC was positively associated with interventricular septum, LV mass index, left atrial volume index, *E*/*e*' ratio, and negatively associated with Tissue Doppler *e*', *E*/*A* ratio (*p* < .05). As a continuous variable, increasing CysC levels were associated with prevalence of LVH (OR: 1.47, 95% confidence interval [CI]: 1.22–1.77), impaired LV diastolic function (OR: 1.58, 95% CI: 1.33–1.87), concentric hypertrophy (OR: 1.54, 95% CI: 1.23–1.93) and eccentric hypertrophy (OR: 1.34, 95% CI: 1.00–1.80) according to multivariate logistic regression analyses. While as a categorical variable, the highest CysC quartile (CysC > 1.04 mg/L) was associated with LVH (OR: 2.95, 95% CI: 1.74–5.00), impaired LV diastolic function (OR: 4.09, 95% CI: 2.54–6.60), and concentric hypertrophy (OR: 3.26, 95% CI: 2.05–5.18).

**Conclusions:**

CysC was significantly associated with early LV remodeling and cardiac functional impairment in T2DM patients with normal renal function. It could be a reliable and convenient biomarker detecting early impairment of cardiac function and structure in T2DM patients.

## 1 INTRODUCTION

Type 2 diabetes mellitus (T2DM) is a systemic endocrine disease that can course multiple organs dysfunction. Cardiovascular diseases (CVDs) and diabetes nephropathy (DN) are the major complications of T2DM with high morbidity and lead to high mortality. Coronary artery disease (CAD) and myocardial infarction are the most severe cardiovascular complications of T2DM resulting in adverse prognostic consequences.^1^ However, T2DM patients are likely to have cardiac remodeling and dysfunction regardless of CAD, especially in the early stage of disease.^2^ Therefore, early detection and intervention of cardiac functional and structural impairment are essential for T2DM patients, otherwise, it will develop into symptomatic heart failure. Furthermore, previous studies demonstrated the differences in biomarker profiles in patients with and without T2DM, indicating the clinical importance to explore the novel biomarkers for early detection of cardiac remodeling and dysfunction in T2DM patients.^3^, ^4^

Cystatin C (CysC), a potent cysteine protease inhibitor that plays pleiotropic roles in human vascular pathophysiology,^5^ is generated by nucleated cells and removed from the bloodstream by the glomeruli. It has been considered to be a reliable biomarker of renal function^6^ and not influenced by age, gender, or muscle mass.^7^, ^8^ Previous studies have suggested that CysC is preferable than creatinine (Cr) for detecting early decrease in renal function, especially for diabetes patients.^9^

Although CysC has already been shown to be a risk factor of CVD morbidity and mortality regardless of patients with nephropathy or not,^10^, ^11^, ^12^ the exact mechanisms remained controversial. In addition, it has been reported that CysC was independently associated with microvascular complications such as diabetic retinopathy and DN in T2DM patients.^13^, ^14^ Diabetes promotes cardiac remodeling partially by cardiac muscle‐specific microvascular complication, thereby aggravating impairment of cardiac function and structure.^15^ These effects occur independent of other structure heart diseases such as CAD and hypertensive heart disease.^2^ Therefore, we hypothesized that CysC is associated with early impairment of cardiac structure and function in T2DM patients. We performed a cross‐sectional study in T2DM patients without overt heart failure and known structural heart diseases to test this hypothesis. To avoid the influence of renal dysfunction, we also excluded the patients with abnormal renal function which was assessed by Cr and Cr‐based estimated glomerular filtration rate (eGFR).

## 2 METHODS

### 2.1 Study population and data collection

This cross‐sectional observational study consecutively enrolled type 2 diabetic patients who admitted in the Third Affiliated Hospital of Sun Yat‐sen University for glycemic control and finished echocardiography examination from April 2019 to May 2020. T2DM was diagnosed according to the 1999 criteria of the World Health Organization (WHO).^16^ Patients were excluded in cases of the following conditions: (1) eGFR <60 ml/min per 1.73 m^2^ which was defined as renal dysfunction^17^; (2) overt heart failure with a New York Heart Association (NYHA) functional classification of III of IV; (3) left ventricular ejection fraction (LVEF) less than 50%; (4) known organic heart diseases including coronary heart disease, valvular heart disease, primary cardiomyopathy, alcoholic cardiomyopathy or congenital heart disease; (5) chronic atrial fibrillation; (6) insufficient clinical data which was essential for our study. Ethical approval was obtained from the Third Affiliated Hospital of Sun Yat‐sen University Network Ethics Committee. Informed consent was obtained from all participants.

### 2.2 Data collection and laboratory measurements

Demographic and clinical data, including gender, age, height, weight, systolic blood pressure (SBP), diastolic blood pressure (DBP), heart rate (HR), lifestyles (smoking status and alcohol consumption), comorbidity, duration of diabetes and medications in the past 2 weeks were obtained for all patients by a well‐trained researcher. Blood pressure and HR were measured in the right upper arm using oscillometric methods after 10 min of rest. Body mass index (BMI) was calculated as body weight divided by the square of height. Hypertension was diagnosed as SBP ≥ 140 mmHg and/or DBP ≥ 90 mmHg or current use of antihypertensive medication.^18^ Blood and urine samples were collected after 8‐h overnight fast. The biochemical parameters including total cholesterol, triglycerides (TG), low‐density lipoprotein cholesterol (LDL‐C), high‐density lipoprotein cholesterol (HDL‐C), blood urea nitrogen, Cr, uric acid (UA), and fasting plasma glucose were examined by HITACHI 7180 automatic‐analyzer. EGFR was calculated using the Modified Diet in Renal Disease (MDRD) formula: eGFR= 186 × (serum Cr^–1.154^) × (age^–0.203^) × 0.742 (if female). HbA1c levels were evaluated by high‐pressure liquid chromatography (Bio‐Rad, D‐10 analyzer). The concentration of serum CysC was measured by particle‐enhanced turbidimetric immunoassay (HITACHI, 7600‐020 autonomic analyzer).

### 2.3 Echocardiography examination

Transthoracic echocardiography (IE33 echocardiography system) was performed on all participants according to the recommendations of the American Society of Echocardiography (ASE) and the European Association of Cardiovascular Imaging (EACI)^19^ by trained, registered cardiac sonographers. The left ventricular internal end‐diastole dimension (LVDd), interventricular septum (IVS), and left ventricular posterior wall thicknesses (LVPW) were measured at the end of left ventricular (LV) diastole, acquired in the parasternal long‐axis view at the level of the mitral valve leaflet tips. The left atrial (LA) volume was measured by the biplane area‐length method using apical four‐ and two‐chamber views at the end‐systolic frame preceding mitral valve opening, and was indexed to body surface area to derive LA volume index (LAVi). Left ventricular mass (LVM) was calculated with the Devereux formula^20^ and normalized by body surface area as left ventricular mass index (LVMI). Left ventricular hypertrophy (LVH) was defined as LVMI > 115 g/m^2^ in men or 95 g/m^2^ in women.^21^ Relative wall thickness (RWT) was calculated by the following formula: RWT = (2 × LVPW)/LVDd. LV geometric patterns representing the LV remodeling were categorized as the following four types according to RWT and LVH: (1) normal geometry, defined as RWT ≤ 0.42 and non‐LVH; (2) concentric remodeling, defined as RWT > 0.42 and non‐LVH; (3) concentric hypertrophy, defined as RWT > 0.42 and LVH; (4) eccentric hypertrophy, defined as RWT ≤ 0.42 and LVH.^22^ LV systolic function was assessed by calculating LVEF with the modified Simpson biplane method. By the pulsed wave Doppler in the apical four‐chamber view, LV inflow was measured. Peak early (*E*) and late (*A*) diastolic velocities and *E*/*A* ratio were obtained afterward. Peak early diastolic mitral annular velocity (*e*') was measured at the junction of the interventricular septum with the mitral annulus in the apical four‐chamber view by pulsed‐wave Tissue Doppler imaging, and the septal *E*/*e*' ratio was calculated. Peak tricuspid regurgitation (TR) systolic jet velocity was assessed with continuous wave Doppler across the tricuspid valve. According to the 2016 ASE/EACI recommendation,^23^ we defined impaired LV diastolic function if two of the following criteria were met: (1) *E*/*e*' ratio > 14; (2) septal *e*' < 7 cm/s; (3) TR velocity > 2.8 m/s; (4) LAVi > 34 ml/m^2^.

### 2.4 Statistical analysis

All statistical analysis was performed by using SPSS 26.0 for Windows (SPSS Inc). Continuous variables were expressed as mean ± SD and categorical variables were presented as frequencies with percentage. Patients were grouped into quartiles according to serum CysC levels (Q1 = the first quartile, CysC ≤ 0.78 mg/L; Q2 = the second quartile, CysC = 0.78–0.89 mg/L; Q3 = the third quartile, CysC = 0.89–1.04 mg/L; Q4 = the fourth quartile, CysC > 1.04 mg/L). Baseline characteristics and echocardiographic parameters were compared among groups using one‐way analysis of variance for continuous variables or Pearson *χ* ^2^ test for categorical variables. We used linear regression analyses to assess the association between CysC levels and cardiac structure and function. Univariate linear regression setting CysC as independent variable and echocardiographic parameters as dependent variables (Model 1) was performed first. Afterward, two multivariate linear regression models were performed to adjust the confounding factors. The first model was adjusted for age, gender, and BMI (Model 2). The second model was additionally adjusted for HR, duration of T2DM, hypertension, smoker, drinker, medications, eGFR, UA, HGB, LDL‐C, HDL‐C, TG, HbA1c (Model 3). In Model 3, we also conducted sensitivity analysis replacing eGFR with Cr in the covariates to compare the different effect of eGFR and Cr. Afterward, univariate and multivariate logistic regression analyses were used to investigate the association between CysC and prevalence of LVH, impaired LV diastolic function, and different LV geometric patterns. Similarly, univariate (Model 1) and two multivariate logistic regression models (Model 2 and Model 3) were performed with the same covariates in linear regression analyses mentioned above. Furthermore, we conducted subgroup analysis stratified by HbA1c levels, presence of hypertension, BMI, gender, age, eGFR, smoking status, alcohol consumption, and tested for potential interactions of these covariates with CysC levels separately. For all tests, a two‐tailed *p* < .05 was considered to indicate statistical significance.

## 3 RESULTS

### 3.1 Participants characteristics

From May 2019 to September 2020, 2038 T2DM patients finished echocardiography examination consecutively enrolled in the study. After exclusion of individuals with known heart diseases, impaired renal function, or missing CysC data, a total of 1135 patients (644 men and 491 women, mean age 58.97 ± 7.25 years) were eligible for analysis (Supporting Information: Figure 1). Clinical characteristics of the subjects are presented in Table 1. Patients with higher serum CysC levels were older, more hypertensive, longer diabetes duration, lower hemoglobin, higher UA, and lower FBG. Of the renal function indices, CysC levels were significantly correlated to Cr, eGFR, and BUN. As for the medications, proportion of ACEI/ARB, CCB, β blocker, and diuretic intakes varied among groups while there were no significant differences of other drugs. However, there were no significant differences of lipid profiles, HbA1c, proportion of smoker, and drinker among groups.

**Table 1:** Baseline characteristics of patients according to different serum CysC quartiles

Variables	Quartile of CysC				*p*‐value
	1	2	3	4	
Quartile range	≤0.78	0.78–0.89	0.89–1.04	＞1.04	‐
*n*	286	283	285	281	‐
Male, *n* (%)	148 (51.7)	171 (60.4)	187 (65.6)	138 (49.1)	<0.001
Age, years	56.47 ± 6.59	57.70 ± 6.95	59.21 ± 6.21	62.54 ± 7.74	<0.001
BMI, kg/m^2^	24.07 ± 3.63	24.90 ± 3.92	24.66 ± 3.46	24.27 ± 3.29	0.025
Hypertension, *n* (%)	101 (35.3)	113 (39.9)	139 (48.8)	170 (60.5)	<0.001
Diabetes duration, years	4.54 ± 5.22	5.45 ± 6.68	6.22 ± 7.00	7.15 ± 7.36	<0.001
Smoking, *n* (%)	58 (20.3)	68 (24.0)	73 (25.6)	62 (22.1)	0.457
Drinking, *n* (%)	37 (12.9)	38 (13.4)	33 (11.6)	30 (10.7)	0.739
SBP, mmHg	128.26 ± 17.77	131.91 ± 18.99	133.27 ± 19.53	135.69 ± 21.45	<0.001
DBP, mmHg	81.69 ± 11.30	81.41 ± 11.46	80.04 ± 12.34	77.58 ± 12.19	<0.001
HR, bpm	83.57 ± 11.82	83.29 ± 12.59	82.74 ± 12.13	82.86 ± 13.92	0.851
Hemoglobin, g/L	137.50 ± 17.25	142.33 ± 19.92	135.73 ± 16.17	127.37 ± 17.96	<0.001
Cr, μmol/L	54.46 ± 13.22	63.44 ± 14.34	71.49 ± 16.28	80.47 ± 17.31	<0.001
eGFR, ml/min/1.73 m^2^	155.67 ± 50.45	128.13 ± 32.70	111.51 ± 31.93	96.48 ± 31.93	<0.001
BUN, μmol/L	4.99 ± 1.30	5.30 ± 1.49	5.73 ± 1.53	6.78 ± 1.85	<0.001
CysC, mg/L	0.68 ± 0.07	0.84 ± 0.03	0.96 ± 0.04	1.27 ± 0.19	<0.001
UA, μmol/L	350.70 ± 102.44	358.28 ± 107.73	379.95 ± 102.70	400.63 ± 104.11	<0.001
TC, mmol/L	4.69 ± 1.39	4.65 ± 1.14	4.52 ± 1.28	4.54 ± 1.30	0.307
TG, mmol/L	1.97 ± 2.69	1.99 ± 2.33	1.81 ± 1.55	1.85 ± 1.26	0.669
LDL‐C, mmol/L	2.95 ± 1.11	3.01 ± 1.04	2.85 ± 1.07	2.85 ± 1.05	0.194
HDL‐C, mmol/L	1.03 ± 0.30	1.01 ± 0.25	1.02 ± 0.29	0.99 ± 0.28	0.338
FBG, mmol/L	9.14 ± 3.91	8.65 ± 4.24	8.19 ± 4.23	8.02 ± 4.28	0.024
HbA1c, %	9.22 ± 2.66	8.87 ± 2.38	8.78 ± 2.42	8.75 ± 2.32	0.077
Medications					
SGLT2 inhibitor, *n* (%)	14 (4.9)	22 (7.8)	30 (10.5)	29 (10.3)	0.131
Statin, *n* (%)	143 (50.0)	153 (54.1)	143 (50.2)	160 (56.9)	0.139
ACEI/ARB, *n* (%)	55 (19.2)	68 (24.0)	92 (32.3)	123 (43.8)	<0.001
β blocker, *n* (%)	16 (5.6)	22 (7.8)	31 (10.9)	41 (14.6)	<0.001
CCB, *n* (%)	31 (10.8)	37 (13.1)	46 (16.1)	83 (29.5)	<0.001
Diuretic, *n* (%)	2 (0.7)	7 (2.5)	10 (3.5)	25 (8.9)	<0.001

*Note*: All data are presented as mean ± SD or *n* (%).

Abbreviations: ACEI, angiotensin‐converting enzyme inhibitors; ARB, angiotensin receptor blocker; BMI, body mass index; BUN, blood urea nitrogen; CCB, calcium channel blocker; Cr, creatinine; CysC, cystatin C; DBP, diastolic blood pressure; eGFR, estimated glomerular filtration rate; FBG, fasting blood glucose; HDL‐C, high‐density lipoprotein cholesterol; HR, heart rate; LDL‐C, low‐density lipoprotein cholesterol; SBP, systolic blood pressure; SGLT2, sodium‐glucose cotransporter 2; TC, total cholesterol; TG, triglyceride; UA, uric acid.

### 3.2 Association between serum CysC levels and cardiac structural and functional parameters

**Figure 1:**
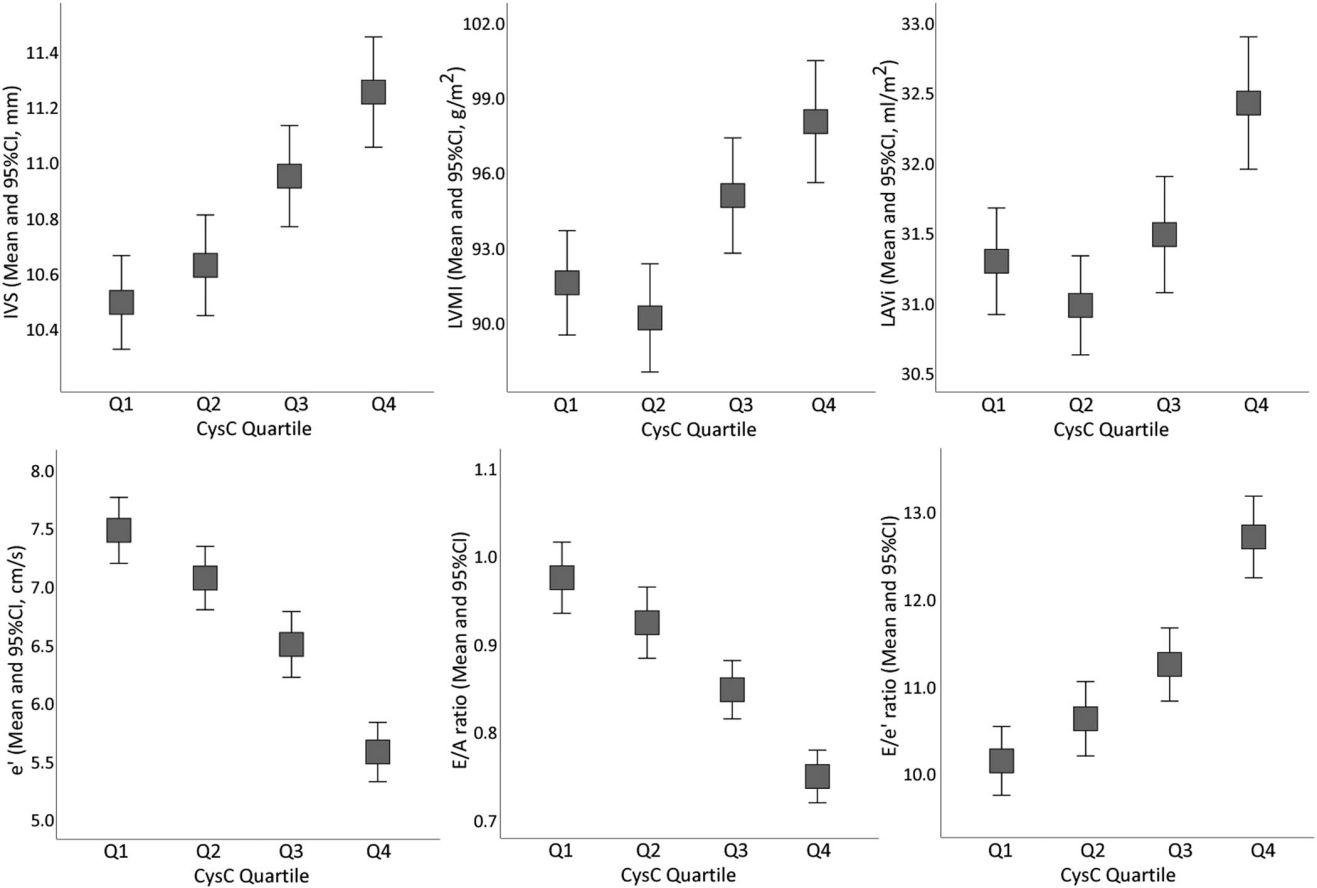
Cardiac structural and functional parameters of patients grouped by quartile of CysC levels. CI, confidence interval; CysC, cystatin C; IVS, interventricular septum; LAVi, left atrial volume index; LVMI, left ventricular mass index.

The echocardiographic parameters reflecting cardiac structure and function were compared among the four CysC quartiles first (Figure 1, Supporting Information: Table 1). In the LV structure changes, increased LVDd, IVS, LVPW, RWT, and LVMI were observed in quartile of higher CysC levels. For parameters reflecting LV diastolic function, patients with higher CysC levels have higher LAVi, *A* velocity, *E*/*e*' ratio, TR velocity and lower *E* velocity, *E*/*A* ratio, Tissue Doppler *e*'. Moreover, LVEF representing LV systolic function was not significantly different among groups.

Univariate and multivariate linear regression analyses were performed afterward to investigate the association between CysC levels and echocardiographic parameters (Table 2, Supporting Information: Figure 2). Univariate analysis (Model 1) demonstrated that CysC levels were positively associated with IVS, LVPW, RWT, LVMI, LAVi, *A* velocity, *E*/*e*' ratio, TR velocity while negatively associated with *E* velocity, *E*/*A* ratio, Tissue Doppler *e*' (*p* <.05). There were no associations between CysC and LVEF, LVDd (*p* > .05). After adjusting for age, gender, and BMI (Model 2), these associations were similar (*p* < .05). Further adjusting for HR, duration of T2DM, hypertension, smoker, drinker, medications, eGFR, UA, HGB, LDL‐C, HDL‐C, TG, HbA1c (Model 3), CysC levels remained associated with IVS, LVMI, LAVi, *A* velocity, *E*/*A* ratio, Tissue Doppler *e*', *E*/*e*' ratio, and TR velocity (*p* < .05).

**Table 2:** Linear regression analysis assessing the relationships of serum CysC levels with left cardiac structural and functional parameters

Echocardiographic parameters	Model 1		Model 2		Model 3	
	*β* (SE)	*p*‐value	*β* (SE)	*p*‐value	*β* (SE)	*p*‐value
LV structure						
LVDd	−0.010 (0.030)	0.741	−0.008 (0.030)	0.787	0.005 (0.033)	0.879
IVS	0.191 (0.029)	<0.001	0.142 (0.029)	<0.001	0.138 (0.034)	<0.001
LVPW	0.093 (0.029)	0.002	0.060 (0.030)	0.046	0.025 (0.036)	0.483
RWT	0.093 (0.028)	0.002	0.067 (0.032)	0.032	0.028 (0.036)	0.458
LVMI	0.167 (0.029)	<0.001	0.121 (0.031)	<0.001	0.123 (0.035)	0.001
LV systolic function						
LVEF	0.007 (0.029)	0.808	−0.005 (0.031)	0.870	0.019 (0.037)	0.606
LV diastolic function						
LAVi	0.149 (0.029)	<0.001	0.090 (0.029)	0.002	0.080 (0.034)	0.017
*E* velocity	−0.108 (0.029)	<0.001	−0.071 (0.032)	0.022	−0.052 (0.038)	0.157
*A* velocity	0.283 (0.029)	<0.001	0.187 (0.028)	<0.001	0.129 (0.033)	<0.001
*E*/*A* ratio	−0.274 (0.029)	<0.001	−0.173 (0.029)	<0.001	−0.115 (0.034)	0.001
Tissue Doppler *e*’	−0.288 (0.030)	<0.001	−0.218 (0.030)	<0.001	−0.194 (0.029)	<0.001
*E*/*e*’ ratio	0.267 (0.029)	<0.001	0.222 (0.030)	<0.001	0.200 (0.035)	<0.001
TR velocity	0.124 (0.030)	<0.001	0.085 (0.029)	0.004	0.117 (0.034)	0.001

*Note*: Model 1 was univariate linear regression analysis. Model 2 was adjusted for age, gender, and BMI. Model 3 was further adjusted for HR, duration of T2DM, hypertension, smoker, drinker, medications, eGFR, UA, HGB, LDL‐C, HDL‐C, TG, HbA1c.

Abbreviations: CysC, cystatin C; eGFR, estimated glomerular filtration rate; HDL‐C, high‐density lipoprotein cholesterol; HR, heart rate; IVS, interventricular septum; LAVi, left atrial volume index; LDL‐C, low‐density lipoprotein cholesterol; LV, left ventricle; LVDd, left ventricular internal end‐diastole dimension; LVEF, left ventricular ejection fraction; LVMI, left ventricular mass index; LVPW, left ventricular posterior wall thicknesses; RWT, relative wall thickness; SE, standard error; TG, triglyceride; TR, tricuspid regurgitation; UA, uric acid; *β*, standardized *β*‐estimates.

For comparison, we also investigated the associations between other renal function indices (eGFR and CR) and echocardiographic parameters (Supporting Information: Table 2). Eventually, eGFR was only found positively associated with IVS and TR velocity, while CR was found uncorrelated with cardiac structural and functional changes.

### 3.3 Association between CysC and LVH, impaired LV diastolic function, LV geometry

In the 1135 T2DM patients without known heart diseases and renal dysfunction, LVH and impaired LV diastolic function were detected in 319 (28.1%) and 348 (30.7%) cases, respectively. LV geometry classified as normal, concentric remodeling, concentric hypertrophy, and eccentric hypertrophy were correspondingly observed in 392 (34.5%), 424 (37.4%), 229 (20.2%), and 90 (7.9%) cases.

**Figure 2:**
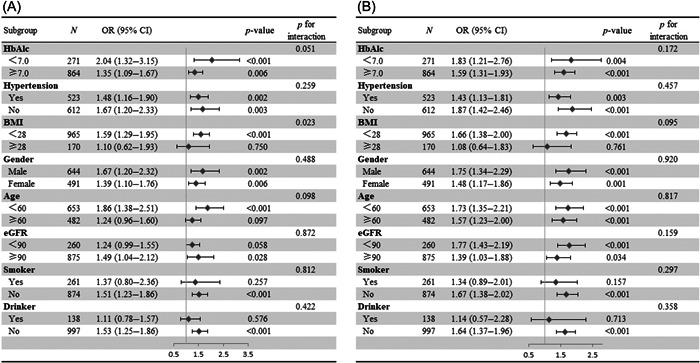
Subgroup analyses for the associations between cystatin C and LVH (A), impaired LV diastolic function (B). BMI, body mass index; CI, confidence interval; eGFR, estimated glomerular filtration rate; LV, left ventricle; LVH, left ventricular hypertrophy; OR, odds ratio.

As a continuous variable, increasing CysC levels were positively associated with both LVH and impaired LV diastolic function after adjusting for all covariates (*p* < .001). Results were similar when we categorized individuals by CysC quartiles: the highest CysC quartile (CysC > 1.04 mg/L) was significantly associated with LVH and impaired LV diastolic function in both unadjusted and adjusted models (*p* < .001) (Table 3). Subgroup analysis showed that CysC levels were significantly associated with LVH in younger patients (age < 60 years) and those with lower BMI (BMI < 28 kg/m^2^), but not in older patients and those with BMI ≥ 28 kg/m^2^ (*p* for interaction <.10). Moreover, the association between CysC and impaired LV diastolic function was existent in the patients with BMI < 28 kg/m^2^ but not in those with BMI ≥ 28 kg/m^2^ (*p* for interaction < .10) (Figure 2).

**Table 3:** Logistic regression analysis assessing the relationships of serum CysC with LV hypertrophy and impaired diastolic function

	Model 1		Model 2		Model 3	
	OR (95% CI)	*p*‐value	OR (95% CI)	*p*‐value	OR (95% CI)	*p*‐value
LVH						
CysC Q1	1.00 (Reference)	‐	1.00 (Reference)	‐	1.00 (Reference)	‐
CysC Q2	1.28 (0.81–2.03)	0.295	1.11 (0.72–1.70)	0.636	0.97 (0.66–1.44)	0.896
CysC Q3	1.63 (1.01–2.65)	0.046	1.39 (0.91–2.14)	0.129	1.09 (0.74–1.60)	0.677
CysC Q4	2.45 (1.70–3.52)	<0.001	2.42 (1.59–3.68)	<0.001	2.95 (1.74–5.00)	<0.001
CysC per 1‐SD	1.48 (1.19–1.68)	<0.001	1.40 (1.21–1.63)	<0.001	1.47 (1.22–1.77)	<0.001
LV impaired diastolic function						
CysC Q1	1.00 (Reference)	‐	1.00 (Reference)	‐	1.00 (Reference)	‐
CysC Q2	1.08 (0.72–1.63)	0.707	1.05 (0.69–1.59)	0.830	1.12 (0.72–1.73)	0.625
CysC Q3	1.60 (1.09–2.37)	0.018	1.53 (1.03–2.28)	0.037	1.53 (0.98–2.38)	0.061
CysC Q4	4.91 (3.38–7.14)	<0.001	4.15 (2.81–6.13)	<0.001	4.09 (2.54–6.60)	<0.001
CysC per 1‐SD	1.78 (1.56–2.04)	<0.001	1.65 (1.43–1.90)	<0.001	1.58 (1.33–1.87)	<0.001
LV geometry						
Concentric remodeling						
CysC Q1	1.00 (Reference)	‐	1.00 (Reference)	‐	1.00 (Reference)	‐
CysC Q2	1.07 (0.73–1.55)	0.738	0.94 (0.64–1.38)	0.765	0.83 (0.56–1.24)	0.366
CysC Q3	1.11 (0.76–1.61)	0.597	0.92 (0.62–1.35)	0.656	0.79 (0.51–1.21)	0.272
CysC Q4	1.43 (0.95–2.16)	0.084	1.17 (0.76–1.79)	0.485	0.95 (0.57–1.58)	0.849
CysC per 1‐SD	1.16 (1.00–1.35)	0.058	1.08 (0.91–1.27)	0.385	1.01 (0.83–1.23)	0.940
Concentric hypertrophy						
CysC Q1	1.00 (Reference)	‐	1.00 (Reference)	‐	1.00 (Reference)	‐
CysC Q2	1.23 (0.71–2.14)	0.466	1.09 (0.65–1.82)	0.755	0.99 (0.61–1.62)	0.977
CysC Q3	1.41 (0.78–2.54)	0.256	1.20 (0.71–2.03)	0.496	1.01 (0.62–1.65)	0.974
CysC Q4	3.68 (1.94–6.98)	<0.001	2.90 (1.74–4.84)	<0.001	3.26 (2.05–5.18)	<0.001
CysC per 1‐SD	1.64 (1.39–1.93)	<0.001	1.48 (1.23–1.77)	<0.001	1.54 (1.23–1.93)	<0.001
Eccentric hypertrophy						
CysC Q1	1.00 (Reference)	‐	1.00 (Reference)	‐	1.00 (Reference)	‐
CysC Q2	1.04 (0.52–2.07)	0.918	1.05 (0.52–2.13)	0.890	1.02 (0.49–2.14)	0.949
CysC Q3	1.47 (0.77–2.82)	0.242	1.60 (0.81–3.14)	0.174	1.47 (0.70–3.09)	0.315
CysC Q4	2.25 (1.16–4.38)	0.016	2.00 (0.99–4.06)	0.054	1.69 (0.72–3.97)	0.230
CysC per 1‐SD	1.51 (1.21–1.88)	<0.001	1.40 (1.11–1.77)	0.005	1.34 (1.00–1.80)	0.048

*Note*: Model 1 was univariate logistic regression analysis. Model 2 was adjusted for age, gender and BMI. Model 3 was further adjusted for HR, duration of T2DM, hypertension, smoker, drinker, medications, eGFR, UA, HGB, LDL‐C, HDL‐C, TG, HbA1c.

Abbreviations: CI, confidence interval; CysC, cystatin C; eGFR, estimated glomerular filtration rate; HDL‐C, high‐density lipoprotein cholesterol; HR, heart rate; LDL‐C, low‐density lipoprotein cholesterol; LV, left ventricle; LVH, LV hypertrophy; OR, odds ratio; TG, triglyceride; UA, uric acid.

We categorized LV geometry as normal geometry, concentric remodeling, concentric hypertrophy and eccentric hypertrophy according to RWT and LVMI. As a continuous variable, increasing CysC levels were positively associated with concentric hypertrophy and eccentric hypertrophy in both unadjusted and adjusted models (*p* < .05). As a categorical variable, highest CysC quartile (CysC > 1.04 mg/L) was significantly associated with both concentric and eccentric hypertrophy (*p* < .05), while it was only correlated to concentric hypertrophy after adjusting for all covariates (*p* < .05) (Table 3).

## 4 DISCUSSION

Our study found the association between CysC and early cardiac structural and functional impairment in T2DM patients with normal renal function and without organic heart diseases. In the echocardiography parameters, elevated serum CysC levels were independently associated with greater IVS, LVMI and LAVi, higher *A* velocity, *E*/*e*' ratio and TR velocity, and lower *E*/*A* ratio and Tissue Doppler *e*'. But CysC was uncorrelated to LVDd and LVEF. Moreover, increased CysC was significantly associated with LVH, impaired LV diastolic function, and LV concentric hypertrophy. These associations were independently of Cr and Cr‐based eGFR, while such relationships were not existent between Cr or eGFR and echocardiography parameters mentioned above.

It has been already established that T2DM resulted in various target organ damage such as DN, diabetic retinopathy, atherosclerosis CVD and diabetic cardiomyopathy. Even in patients with prediabetes, the risk of CVD and heart failure were significantly increased.^24^, ^25^ Thus, T2DM patients have greater rate of development to fatal cardiovascular events, especially those without early detection and well controlled.^26^ In addition, previous studies have already demonstrated that diabetes promoted cardiac remodeling which was significantly associated with cardiac structural and functional impairment.^2^ These changes occurred independently of other cardiac risk factors such as CAD and hypertension, and might further lead to the progression of diabetic cardiomyopathy and clinical heart failure.^27^ These studies highlighted the importance of early realization and prevention of cardiac remodeling and dysfunction in patients with impaired glucose regulation. In consistent with previous studies, our cross‐sectional study excluding patients with overt heart failure and organic heart diseases also found a high prevalence of subclinical cardiac remodeling and LV diastolic dysfunction in T2DM patients. Furthermore, elevated serum CysC levels were found associated with these cardiac abnormalities, indicating that CysC could be a marker help detecting the subclinical cardiac remodeling in T2DM patients.

Prospective epidemiological studies have already demonstrated that CysC was an independent risk factor of future CVD occurrence and progression.^28^, ^29^ Previous cross‐sectional studies also found the association between serum CysC levels and cardiac structural or functional abnormalities.^30^, ^31^, ^32^ However, these studies did not exclude subjects with chronic kidney diseases. Recently, CysC was considered to be a more reliable biomarker of renal function rather than Cr especially in some particular situations.^6^, ^7^, ^8^ As it was quite clear that renal dysfunction could cause cardiac structural abnormality and functional damage,^33^, ^34^ the results from previous studies might be partially explained by the correlation between renal dysfunction and cardiac remodeling. Hence, we conducted this study excluding the patients with elevated Cr or eGFR < 60 ml/min per 1.73 m^2^ to avoid the confounding of moderate to severe renal impairment. Eventually, the associations between CysC and cardiac structural and functional abnormality were still existent in patients with normal renal function. In addition, this is the first study to demonstrate these associations in T2DM patients without heart failure and structural heart disease. As T2DM is the most important risk factor of preclinical cardiac dysfunction progression,^35^ these results provide important clinical value for CysC in detecting early impairment of cardiac function and structure for T2DM patients without both renal dysfunction and clinical heart failure, thereby to formulate early intervention and prevent cardiac dysfunction development.

Although CysC has been proven to be an independent risk factor of CVD, the mechanisms still remained controversial. Van der Laan SW et al performed a Mendelian randomization study^5^ to investigate the causal relationship between CysC and CVDs including CAD, ischemic stroke, and heart failure in general population. Eventually, the results did not support a causal role of CysC in the etiology of CVD. Therefore, we tend to consider CysC as a marker rather than a maker of CVD. The association between serum CysC levels and cardiac abnormality in T2DM patients could be explained by the similar mechanisms.

First, as mentioned above, CysC has been proved to be a reliable biomarker of renal function and was more sensitive than Cr in the early stage of renal dysfunction. Similarly, previous studies also suggested that CysC could be a preferable marker in early recognition of diabetic nephropathy. Although we have excluded the patients with decreased eGFR and elevated Cr, a subset of the patients included in our study still might have early diabetic kidney damage. Our study has already excluded the patients with heart failure and structural heart diseases, thus the impairment of cardiac structure and function could be mainly attributed to diabetes itself which was considered to be the early stage of diabetic cardiomyopathy. As microvascular complications of T2DM, there were many common pathophysiological mechanisms of diabetic nephropathy and diabetic cardiomyopathy.^36^ Therefore, CysC was elevated in patients with early diabetic kidney damage and these patients might also have early diabetic myocardial damage.

Second, microvessels and myocardia impaired by diabetes might secrete more CysC into the circulation. Previous studies have already suggested that tissue CysC levels were reduced in atherosclerotic plaques while cells outside of the vascular walls secrete more CysC into the circulation.^37^, ^38^, ^39^ Moreover, a recent study found serum CysC levels were higher in patients with LVH. Afterward the authors investigated the mechanisms by animal models and eventually they found cardiomyocyte could secrete more CysC which was induced by pressure overload.^40^ Thus, in our study, the damaged microvessels and myocardia might have increased secretion of cystatin C, which can partially explain their correlation.

In addition, although Mendelian randomization study did not find a causal role of CysC in the etiology of CVD, basic research still showed the direct effect of CysC in cardiovascular pathophysiological changes. As an endogenous inhibitor of cysteine protease, the imbalance between cysteine proteases and CysC was considered to be implicated in the pathogenesis of cardiac remodeling.^41^, ^42^, ^43^

There were some limitations in our study. First, as a cross‐sectional study in single center, the sample size was relatively small. Second, considering the economic feasibility and clinical applicability, we evaluated cardiac structure and function using transthoracic echocardiography rather than cardiac MRI. Although we have already included indices reflecting cardiac function as comprehensive as possible, we did not have the cardiac mechanics indices such as global longitudinal strain, which could help to evaluate cardiac function more precisely. Third, our study excluded the patients with known organic heart diseases. Except CAD, other structural heart diseases could be diagnosed by echocardiography. However, definite diagnosis of CAD required coronary computed tomography angiography or coronary angiography. Most patients in our study finished these examinations, but there were still a small number of patients diagnosed CAD through clinical symptoms, electrocardiogram, and echocardiography. Finally, this is a cross‐sectional study without follow‐up for the future changes of cardiac structure and function. Although our results have provided evidence that CysC could be a marker detecting the early impairment of cardiac structure and function in T2DM patients, the causality relationship between CysC and LV remodeling and diastolic dysfunction cannot be determined. Thus prospectively cohort study was expected to investigate the effects of CysC in the future cardiac structural and functional changes.

## 5 CONCLUSION

In summary, our study demonstrated that elevated serum CysC was significantly associated with early LV remodeling and cardiac functional impairment in T2DM patients with normal renal function, mostly reflecting in LVH, impaired LV diastolic function, and LV concentric hypertrophy. These associations indicated that CysC could be a reliable and convenient biomarker detecting early impairment of cardiac function and structure in T2DM patients.

## AUTHOR CONTRIBUTIONS

Xinxue Liao and Xiaodong Zhuang designed the study and were in charge of the overall direction and planning. Zhuoshan Huang analyzed the data and wrote Abstract, Introduction, Results, and Discussion parts of the manuscript with input from all the authors. Junlin Zhong was responsible for echocardiography examination and wrote Methods part of the manuscript. Shaozhao Zhang and Zhenyu Xiong performed the statistical analyses. Yiquan Huang and Menghui Liu prepared figures and tables. Zhuoshan Huang, Junlin Zhong, and Xiaomin Ye collected the data needed for our study. Yifen Lin and Xiangbin Zhong were responsible for the modification of manuscript.

## CONFLICT OF INTEREST

The author declares no conflict of interest.

## Supporting information

Supporting information.

Supporting information.

Supporting information.

Supporting information.

## Data Availability

The datasets used and/or analyzed during the current study are available from the corresponding author on reasonable request.
