# Supplementary figures and images for: Association between serum cystatin C and early impairment of cardiac function and structure in type 2 diabetes patients with normal renal function
Source: Clin Cardiol. 2022 Sep 14;45(12):1287–96. doi: 10.1002/clc.23920 (PMC9748767; doi:10.1002/clc.23920)

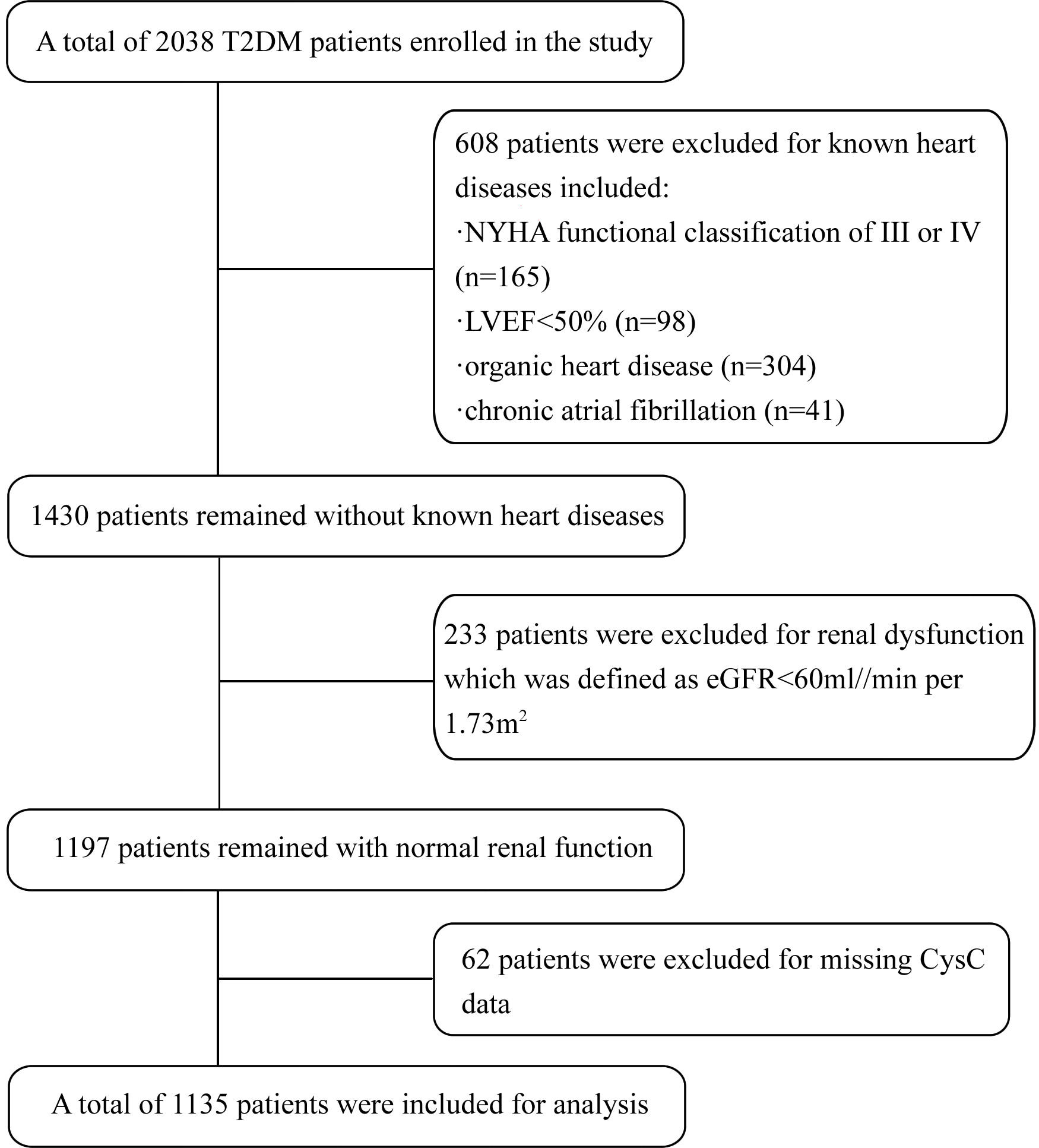

Supplement: Supplementary file 1 — Supporting information. [file CLC-45-1287-s004.jpg]

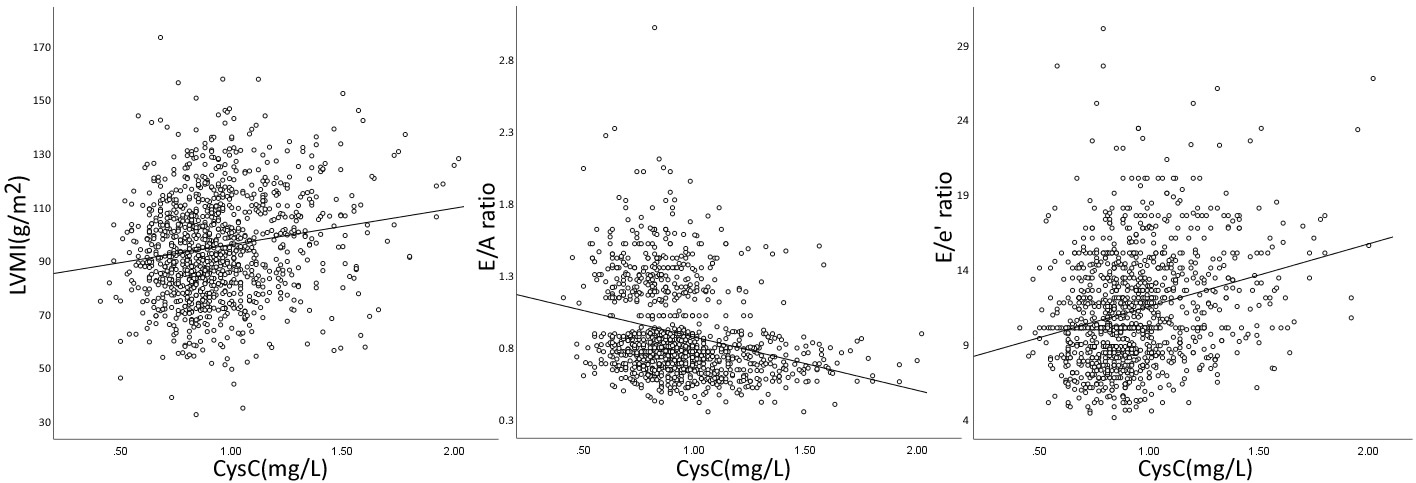

Supplement: Supplementary file 2 — Supporting information. [file CLC-45-1287-s002.jpg]
