# Supplementary material for: Association between serum cystatin C and early impairment of cardiac function and structure in type 2 diabetes patients with normal renal function
Source: Clin Cardiol. 2022 Sep 14;45(12):1287–96. doi: 10.1002/clc.23920 (PMC9748767; doi:10.1002/clc.23920)
Supplement: Supplementary file 3 — Supporting information. [file CLC-45-1287-s003.docx]

Supplementary Table 1 Cardiac structural and functional parameters of patients grouped by quartile of CysC levels

|  | | Quartile of CysC | | | | | | P value |
| --- | --- | --- | --- | --- | --- | --- | --- | --- |
|  |  | 1 | 2 | | 3 | | 4 |  |
| **LV structure parameters** |  | | |  | |  | |  |
| LVDd,mm | | 43.88±4.13 | 44.13±3.86 | | 44.41±3.43 | | 44.92±3.92 | 0.009 |
| IVS,mm | | 10.50±1.44 | 10.63±1.54 | | 10.95±1.55 | | 11.25±1.68 | <0.001 |
| LVPW,mm | | 9.49±1.09 | 9.51±1.00 | | 9.59±1.12 | | 9.74±1.11 | 0.023 |
| RWT | | 0.43±0.05 | 0.43±0.07 | | 0.43±0.06 | | 0.45±0.06 | <0.001 |
| LVMI,g/m^2^ | | 91.58±17.92 | 90.18±18.48 | | 95.07±19.76 | | 98.03±20.77 | <0.001 |
| **LV systolic function parameter** | | | |  | |  | |  |
| LVEF,% | | 67.06±4.82 | 67.17±4.63 | | 66.95±4.85 | | 66.88±4.94 | 0.896 |
| **LV diastolic function parameters** | | | |  | |  | |  |
| LAVi, ml/m^2^ | | 31.29±3.27 | 30.98±3.02 | | 31.48±3.56 | | 32.42±4.02 | <0.001 |
| E velocity,m/s | | 0.71±0.18 | 0.69±0.16 | | 0.68±0.16 | | 0.65±0.16 | 0.001 |
| A velocity,m/s | | 0.76±0.18 | 0.80±0.20 | | 0.83±0.19 | | 0.90±0.20 | <0.001 |
| E/A ratio | | 0.98±0.35 | 0.92±0.35 | | 0.85±0.28 | | 0.75±0.25 | <0.001 |
| Tissue Doppler e’,cm/s | | 7.47±2.43 | 7.07±2.32 | | 6.50±2.41 | | 5.58±2.17 | <0.001 |
| E/e’ ratio | | 10.15±3.38 | 10.63±3.63 | | 11.25±3.58 | | 12.71±3.97 | <0.001 |
| TR velocity, cm/s | | 2.52±0.30 | 2.58±0.32 | | 2.60±0.30 | | 2.63±0.34 | <0.001 |
| **LVH, n(%)** | | 66(23.1) | 64(22.6) | | 70(24.6) | | 119(42.3) | <0.001 |
| **LV impaired diastolic function, n(%)** | | 56(19.6) | 59(20.8) | | 80(28.1) | | 153(54.4) | <0.001 |
| **LV geometry** | |  |  | |  | |  | <0.001 |
| Normal, n(%) | | 112(39.2) | 108(38.2) | | 104(36.5) | | 68(24.2) |  |
| Concentric remodeling, n(%) | | 108(37.8) | 111(39.2) | | 111(38.9) | | 94(33.5) |  |
| Concentric hypertrophy, n(%) | | 47(16.4) | 45(15.9) | | 44(15.4) | | 93(33.1) |  |
| Eccentric hypertrophy, n(%) | | 19(6.6) | 19(6.7) | | 26(9.1) | | 26(9.3) |  |

All data are presented as mean±SD or n (%).

LV, left ventricle; LVDd, left ventricular internal end-diastole dimension; IVS, interventricular septum; LVPW, left ventricular posterior wall thicknesses; RWT, relative wall thickness; LVMI, left ventricular mass index; LVEF, left ventricular ejection fraction; LAVi, left atrial volume index; TR, tricuspid regurgitation; LVH, LV hypertrophy.
