# Supplementary material for: Association between serum cystatin C and early impairment of cardiac function and structure in type 2 diabetes patients with normal renal function
Source: Clin Cardiol. 2022 Sep 14;45(12):1287–96. doi: 10.1002/clc.23920 (PMC9748767; doi:10.1002/clc.23920)
Supplement: Supplementary file 4 — Supporting information. [file CLC-45-1287-s001.docx]

Supplementary Table 2 Multiple linear regression analysis assessing the relationships of renal function indices with left cardiac structural and functional parameters

| Echocardiographic parameters | | eGFR* | |  | Cr** | |  | CysC** | |
| --- | --- | --- | --- | --- | --- | --- | --- | --- | --- |
|  |  | β(SE) | P value |  | β(SE) | P value |  | β(SE) | P value |
| **LV structure** | | |  |  |  |  |  |  |  |
| LVDd | 0.042(0.032) | | 0.265 |  | -0.032(0.041) | 0.432 |  | 0.004(0.041) | 0.922 |
| IVS | 0.075(0.025) | | 0.040 |  | -0.049(0.033) | 0.092 |  | 0.146(0.036) | <0.001 |
| LVPW | -0.022(0.022) | | 0.567 |  | 0.049(0.033) | 0.226 |  | 0.008(0.040) | 0.839 |
| RWT | -0.049(0.034) | | 0.223 |  | 0.058(0.036) | 0.182 |  | 0.018(0.036) | 0.647 |
| LVMI | 0.075(0.039) | | 0.053 |  | -0.067(0.041) | 0.107 |  | 0.126(0.038) | 0.001 |
| **LV systolic function** | | |  |  |  |  |  |  |  |
| LVEF | | 0.044(0.035) | 0.279 |  | -0.050(0.046) | 0.252 |  | 0.027(0.041) | 0.510 |
| **LV diastolic function** | | |  |  |  |  |  |  |  |
| LAVi | | 0.046(0.035) | 0.210 |  | -0.076(0.041) | 0.053 |  | 0.100(0.036) | 0.005 |
| E velocity | | -0.014(0.032) | 0.721 |  | 0.014(0.033) | 0.745 |  | -0.053(0.040) | 0.179 |
| A velocity | | -0.043(0.034) | 0.225 |  | 0.039(0.035) | 0.299 |  | 0.127(0.035) | <0.001 |
| E/A ratio | | 0.032(0.036) | 0.373 |  | -0.033(0.040) | 0.388 |  | -0.111(0.036) | 0.002 |
| Tissue Doppler e’ | | -0.071(0.034) | 0.055 |  | 0.024(0.031) | 0.542 |  | -0.175(0.039) | <0.001 |
| E/e’ ratio | | 0.047(0.036) | 0.212 |  | -0.010(0.036) | 0.798 |  | 0.185(0.038) | <0.001 |
| TR velocity | | 0.100(0.043) | 0.007 |  | -0.068(0.040) | 0.090 |  | 0.109(0.037) | 0.003 |

eGFR, estimated glomerular filtration rate; Cr, creatinine; β, standardized β-estimates; SE, standard error; LV, left ventricle; LVDd, left ventricular internal end-diastole dimension; IVS, interventricular septum; LVPW, left ventricular posterior wall thicknesses; RWT, relative wall thickness; LVMI, left ventricular mass index; LVEF, left ventricular ejection fraction; LAVi, left atrial volume index; TR, tricuspid regurgitation.

*Results from multiple linear regression analysis model 2 which included covariates of age, gender, BMI, HR, duration of T2DM, hypertension, smoker, drinker, medications, UA, HGB, LDL-C, HDL-C, TG, HbA1C, CysC and eGFR.

**Results from sensitivity analysis replacing eGFR with Cr in model 2.
